# Arylesterase Phenotype-Specific Positive Association Between Arylesterase Activity and Cholinesterase Specific Activity in Human Serum

**DOI:** 10.3390/ijerph110201422

**Published:** 2014-01-27

**Authors:** Yutaka Aoki, Kathy J. Helzlsouer, Paul T. Strickland

**Affiliations:** 1 Department of Environmental Health Sciences, Johns Hopkins Bloomberg School of Public Health, Baltimore, MD 21205, USA; E-Mail: pstrickl@jhsph.edu; 2 Department of Epidemiology, Johns Hopkins Bloomberg School of Public Health, Baltimore, MD 21205, USA; 3 Mercy Medical Center, Baltimore, MD 21202, USA; E-Mail: khelzlsouer@mdmercy.com

**Keywords:** pseudocholinesterase, arylesterase, paraoxonase, genetic polymorphism, biomarkers, internal dose, albumin

## Abstract

*Context*: Cholinesterase (ChE) specific activity is the ratio of ChE activity to ChE mass and, as a biomarker of exposure to cholinesterase inhibitors, has a potential advantage over simple ChE activity. *Objective*: To examine the association of several potential correlates (serum arylesterase/paraoxonase activity, serum albumin, sex, age, month of blood collection, and smoking) with plasma ChE specific activity. *Methods*: We analyzed data from 195 cancer-free controls from a nested case-control study, accounting for potential confounding. *Results*: Arylesterase activity had an independent, statistically significant positive association with ChE specific activity, and its magnitude was the greatest for the arylesterase phenotype corresponding to the QQ PON1_192_ genotype followed by phenotypes corresponding to QR and RR genotypes. Serum albumin was positively associated with ChE specific activity. *Conclusions*: Plasma arylesterase activity was positively associated with plasma ChE specific activity. This observation is consistent with protection conferred by a metabolic phenotype resulting in reduced internal dose.

## 1. Introduction

Inhibition of cholinesterase (ChE) activity in blood has been used as a biomarker for exposure to organophosphates (OPs) and carbamates since 1950 [1]. Human plasma contains butyryl cholinesterase (also called pseudocholinesterase and referred to simply as cholinesterase or ChE in this article), which is inhibited by organophosphates and carbamates, widely-used classes of insecticides. ChE inhibition has been used to confirm or detect exposure to cholinesterase inhibitors.

Because of the large inter-individual variability in the mass concentration of ChE in plasma, a single measurement of ChE activity alone cannot accurately detect inhibition in an individual. An approach to potentially overcome this limitation is to measure ChE mass, which is the concentration of ChE enzyme measured typically by enzyme-linked immuno-absorbent assay (ELISA), in blood along with ChE activity and calculate “specific ChE activity” (activity divided by mass).

The idea to use ChE specific activity as a marker of exposure to ChE inhibitors is not new. Whittaker [2] promoted it citing a publication on an immunoassay for ChE mass [3]. Despite the promise of improved utility as an exposure marker, only a limited number of studies have evaluated ChE specific activity [4,5]. Most studies on ChE and exposure to cholinesterase inhibitors have been conducted using ChE activity alone and thus our knowledge of the factors (e.g., sex, age, and season) that may influence ChE specific activity and ChE mass is limited.

Exposure to cholinesterase-inhibiting insecticides is ubiquitous at present and has been so in the past few decades. Data from National Health and Nutrition Survey (NHANES) for 1999–2001 show that the majority of individuals in the U.S. have detectable levels of organophosphate and carbamate metabolites in urine [6]. Two major routes of exposure to organophosphates for the general population were deemed to be ingestion of contaminated food and hand-to-mouth contact with surfaces with organophosphate residues [6]. The latter would mostly result from residential indoor pesticide application, for which chlorpyrifos and diazinon were two major organophosphates used. Residential indoor use of these two pesticides was largely phased out by mid-2000s following implementation of Food Quality Protection Act of 1996 [7]. The absence of biomonitoring data such as NHANES for the earlier decades makes it difficult to estimate exposure levels in the past. Nonetheless, judging from the general decline in organophosphate use since 1980 [8] and the recent phase-out of residential indoor use, the general population presumably had higher exposure in the 1970s and 1980s than in recent years.

This report presents results of an investigation into the association between ChE specific activity and several factors including ChE phenotypes, serum paraoxonase phenotype and activity, ChE mass concentration, serum albumin, month of blood collection, age, and sex, using blood samples collected from apparently-healthy individuals in general population. Animal prophylactic experiment results [9] and human observational findings from occupational settings with substantial exposure to OPs [10] support the notion that low serum paraoxonase activity is a metabolic susceptibility factor for OP intoxication. The main purpose of this study was to seek evidence for paraoxonase phenotype and activity as a susceptibility factor by examining how they are associated with ChE specific activity, which is a marker of internal dose of cholinesterase inhibitors, in the general population. PON1 192 genotype influences catalytic efficiency, and accounting for both the genotype and quantity of serum paraoxonase, which we approximate using arylesterase phenotype and activity, is important in studying paraoxonase as susceptibility factor for OP intoxication [10]. Association between serum albumin and ChE specific activity also was studied since it may influence ChE activity and its measurement due to its potential inhibitory or stimulatory effects on ChE specific activity (see Discussion). Other factors as potential causes of variation in ChE specific activity were investigated to address existing knowledge gaps regarding whether some previously reported associations between certain factors (e.g., season and sex) and ChE activity are attributable to their association with ChE mass or ChE specific activity. This study used data collected from the cancer-free controls of a case-control study of non-Hodgkin’s lymphoma [11].

## 2. Experimental Section

### 2.1. Design

This study used data from only controls of a nested case-control study, preliminary results of which were reported elsewhere [11]. Overall design of the study can be characterized as a variation of cross-sectional study in which data collected at two time points are used. The parent nested case-control study employed a 1:2 matching scheme, which was described in detail in previously published studies that used the same set of cases and controls [12,13]. While this article is based on the data from the controls only, the laboratory analyses were performed in a masked manner in terms of case-control status of the samples, and so reference to the case samples are occasionally made in this section. The main dependent variable of this study was ChE specific activity, and describing its association with other variables was the primary aim of this investigation. This study was approved by the Committee on Human Research at Johns Hopkins Bloomberg School of Public Health. All participants provided written informed consent at the time of entry into the cohort. More detailed method description is given elsewhere [11].

### 2.2. Subjects

Data from the controls of the parent case-control study were used to preclude observing associations arising from the disease development processes; we were interested in factors that influence ChE specific activity among apparently healthy subjects. These subjects took part in either or both of the CLUE I and CLUE II cohort studies conducted in Washington County (MD, USA) in 1974 and 1989, respectively. They donated blood and answered a brief questionnaire. All samples were kept frozen at −70 °C until thawed to be aliquoted for this study and further stored at −50 °C until realiquoted to be assayed. Sera from the CLUE I cohort and heparinized plasma from the CLUE II cohort were used. Serum for one eligible control was not properly retrieved. Consequently, 113 subjects from “CLUE I only” participants, 24 subjects from “CLUE II only” participants, and 58 subjects from “CLUE I & II” participants were included in this investigation. Serum and plasma from a voluntary donor of usual-usual (UU) ChE phenotype with no known substantial exposure to cholinesterase inhibitors were collected on a single occasion, stored at −50 °C, and used as standards for the entire investigation.

### 2.3. Assays

The levels of cholinesterase activity and immunoreactive mass are expressed relative to those of the corresponding standard serum or plasma. Cholinesterase activity was measured by an Ellman-type microplate kinetic assay using butyrylthiocholine iodide as a substrate and 6,6'-dithiodinicotinic acid as a chromogen [14,15]. In order to minimize the influence of cholinesterase reactivation, the reaction was started by adding substrate-chromogen mixture (200 μL) to an undiluted sample (1.5 μL). Substrate blanks (sample or standard plus chromogen, *i.e.*, the full mixture minus substrate) were run, and their activity subtracted from sample/standard activity.

Immunoreactive cholinesterase mass was measured using a sandwich ELISA. 100 μL of monoclonal antibody (HAH002-0-1, AntiBodyShop, Copenhagen, Denmark, 1:12,000 dilution in 0.01 M phosphate buffer pH 7.2) was used to coat each well of a microplate (Immulon HBX4, Thermo Labsystems, Milford, MA, USA) for 24 h on wet ice. To prevent drying and loss of antigen binding, a small amount of wash buffer was left in coated wells, which were covered with Mylar tape and kept frozen at −150 °C. Phosphate buffer (0.01 M pH 7.2) with 0.2% Tween 20 was used as a diluent and washing buffer for the rest of the ELISA. Samples diluted 1:1,600 and serially-diluted standards (100 μL) were placed in wells. Samples were arranged in triads, each containing samples from a case and two-matched controls. In order to minimize measurement error due to a spatial gradient in binding efficiency within plate, microplate wells were grouped into 6 blocks of 16 (4 × 4) wells and each set of 3 samples was placed in the same block using a version of Latin square design along with standards. Placement patterns were changed across blocks such that the influence of the spatial gradient on the signal-standard level relationship was minimized. 

In order to minimize error for ChE specific activity measurements, assays were repeated for any triad involving a sample with a coefficient of variation greater than 10%. The coefficient of variation was calculated for a pair of specific activity means, each based on measurements in octuplicate. About a third of all samples were re-assayed, and 6% were analyzed for a third time.

ChE phenotypes were determined using benzoylcholine as a substrate and four differential inhibitors: pancuronium bromide dibutylate analogue [16]; urea [17]; succinylcholine and dibucaine [18]. Four ChE phenotypes were identified: usual-usual (UU); usual-atypical (UA), usual-fluoride resistant (UF); atypical-atypical (AA). For all 58 pairs of sera and plasmas from the same individuals collected in 1974 and 1989, respectively, identical ChE phenotype results were obtained for the serum and plasma.

As close surrogates for paraoxonase activity and genotypes, arylesterase activity and phenotypes were determined by the method of Haagen and Brock [19] adapted for microplate. In this method, activity towards *p*-nitrophenyl acetate (0.5 mM) was measured in the absence or the presence of phenyl acetate (1 mM), an inhibitor. The assay solution also included 25 mM triethanolamine hydrochloride, 1 mM CaCl2 (essential in preserving arylesterase catalytic activity) and 1.0 M NaCl. All arylesterase measurements were made within the linear range. Samples with initial measurement greater than 100 mOD/min, which showed nonlinear change in absorbance over time, were diluted 1:2 or 1:3 and re-assayed such that diluted sample’s activity was measured in the linear range. Resultant measurements were scaled back to represent the value corresponding to the undiluted samples. A plot of inhibited activity and non-inhibited activity was used to group samples into three phenotypes designated as G1, G2, and G3 by Haagen and Brock [19], which correspond to QQ, QR, and RR paraoxonase (PON1_192_) genotypes. Of 58 pairs of sera and plasmas from the same controls collected in 1974 and 1989, respectively, there was no disagreement between serum- and plasma-based PON1_192_ phenotyping results. Albumin was measured by a modified bromocresol purple dye-binding method [20].

### 2.4. Statistical Analysis

Rates of absorbance development were used as enzyme activity/mass measures. Each control pair was matched on age, race, date of blood donation, and participation in CLUE studies and censuses. As a measure of reliability, intraclass correlation coefficients were calculated for CLUE I sera, CLUE II plasmas, and the two combined, treating each sample for which duplicate measurements were made as a unit of analysis. This analysis entailed treating all samples as independent from each other, but in reality there were some correlation among samples arising from matching (and, for the analysis based on both CLUE I sera and CLUE II plasmas, from repeated measurements for the same individual). Nonetheless, the intraclass correlation coefficients derived in this manner serve as an interpretable measure of relative size of within-sample variation compared to observed variation across the samples that we aimed to differentiate quantitatively. Skewness of specific activity was tested separately for CLUE I sera and CLUE II plasmas also ignoring matching. The main analysis of association between a dependent variable (ChE specific activity) and covariates was performed using Generalized Estimating Equation (GEE) models with equal correlation structure in order to account for matching. All *p*-values were based on a Wald test for a (set of) regression coefficient(s). The proportion of variation explained by a subset of covariates in a model was estimated using marginal R-squared [21] by fitting two models, one with the covariates of interest and the other without, calculating marginal R-squared for each of them, and taking the difference between the two marginal R-squared. We will call the quantity partial marginal R-squared. Stata statistical package version 8 [22] was used for all analyses and graphics.

## 3. Results and Discussion

### 3.1. Results

#### 3.1.1. Subject and Sample Description

**Figure 1:**
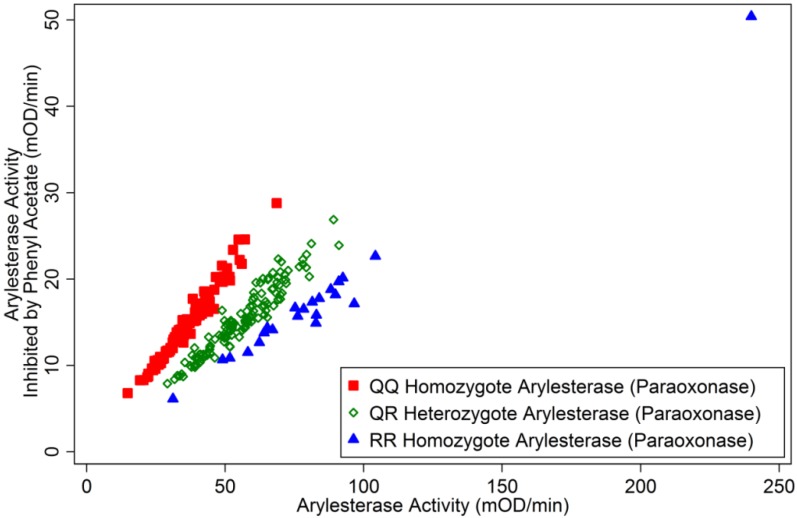
Arylesterase activity (uninhibited *vs*. inhibited by phenyl acetate) by QQ, QR, and RR paraoxonase (PON1_192_) phenotypes.

**Figure 2:**
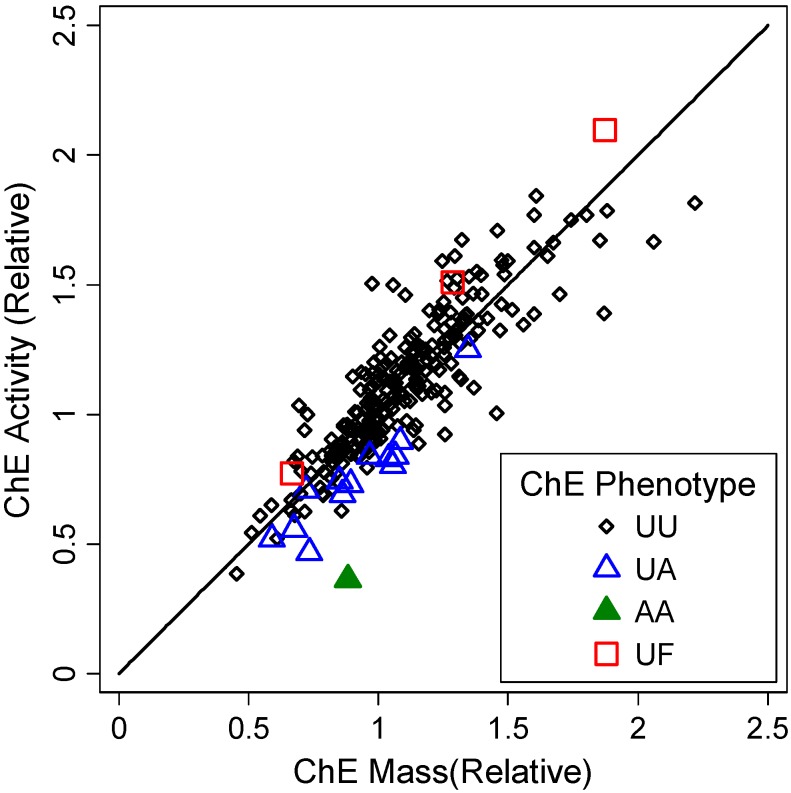
ChE activity and mass by ChE phenotype with the diagonal line representing a theoretical equation, (ChE activity) = (ChE mass), for hypothetical data points with the same ChE specific activity as the standard.

Characteristics of study subjects are summarized in Table 1. Figure 1 shows the plot of uninhibited *vs*. inhibited arylesterase activities, demonstrating reasonably clear separation of phenotypes. In Figure 2, ChE activity is plotted against ChE mass. The data points tend to cluster near the diagonal line for relative activity = relative mass (*i.e.*, specific activity being equal to that of the standard sera or plasma). The slope of the line connecting each data point and the origin in Figure 2 is the specific activity.

Observed phenotype proportions were as follows: for ChE, UU (92.8%), UA (5.1%), AA (0.5%), UF (1.5%); and for arylesterase/paraoxonase, QQ (43.6%), QR (47.2%), RR (9.2%). Similar values were previously reported for Caucasians [10].

Intraclass correlation coefficients for ChE specific activity of CLUE I sera, CLUE II plasmas, and sera/plasmas combined were 0.82, 0.62, and 0.78, respectively, demonstrating good-to-excellent reproducibility.

**Table 1:** Characteristics of research subjects.

Characteristic	CLUE I		CLUE II	
	%	(n ^a^)	%	(n)
Sex: Male	50	(86)	49	(40)
Female	50	(85)	51	(42)
Race: White	98	(168)	99	(81)
Black	2	(3)	1	(1)
Smoking status:Never	40	(69)	37	(30)
Former	29	(49)	51	(42)
Current	31	(53)	12	(10)
Education: <12 years	50	(85)	40	(33)
≥12 years	50	(86)	60	(49)
	Mean	(SD)	Mean	(SD)
Age at blood donation (years)	52.7	13.3	63.1	11.8
ChE Raw activity (unitless ^b^)	1.11	0.31	1.10	0.25
ChE Mass (unitless)	1.10	0.30	1.07	0.21
ChE Specific activity (unitless)	1.01	0.15	1.03	0.10
Arylesterase activity (mOD/min):				
QQ phenotype	37	10	34	8
QR phenotype	59	13	51	12
RR phenotype	84	44 ^c^	77	16
Albumin (g/dL)	5.1	0.7	4.6	0.48

^a^ 58 subjects took part in both CLUE I and CLUE II; ^b^ ChE raw activity, mass, and specific activity are expressed as a unitless number relative to the level measured in a standard (serum or plasma); ^c^ This group included an observation with an exceptionally high arylesterase activity, removal of which gives mean and SD of 74 and 19, respectively.

**Figure 3:**
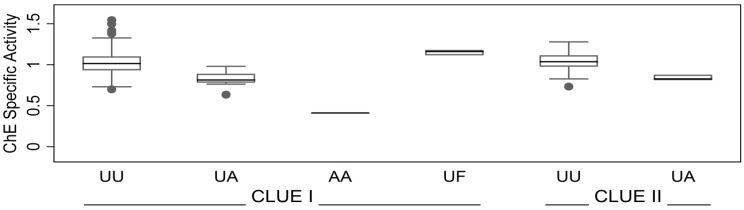
ChE specific activity by sample source (CLUE I or II) and ChE phenotype. UU, usual/usual; UA, usual/atypical; AA, atypical/atypical; UF, usual/fluoride-resistant. ChE specific activity is expressed as a unitless number in relation to the level in the standard.

ChE specific activity levels varied by ChE phenotypes (Figure 3). Change of an allele from usual to atypical was associated with a decrease in ChE specific activity: mean decreases were 0.22 (*p* = 2 × 10^−10^) and 0.19 (*p* = 0.0003) for CLUE I sera and CLUE II plasmas, respectively. This confirms the well-known feature of the atypical ChE, *i.e.*, reduced substrate affinity [2]. For rare fluoride-resistant/usual (UF) heterozygotes ChE specific activity was higher by 0.15 (*p* = 0.01) for CLUE I sera (no UF found in CLUE II plasmas) than for the usual homozygotes. Distribution of specific activity of UU ChE phenotype was: right-skewed for CLUE I sera (*p* = 0.0001); symmetrical for CLUE II plasmas (*p* = 0.29). With adjustment for ChE phenotype, means for specific activities were not different (*p* = 0.35) between CLUE I sera and CLUE II plasmas. Eighteen % of CLUE I sera and 15% of CLUE II plasmas had 90% or lower specific activity (greater than 10% inhibition) compared to the ChE phenotype-specific mean.

#### 3.1.2. Correlates of ChE Specific Activity

**Figure 4:**
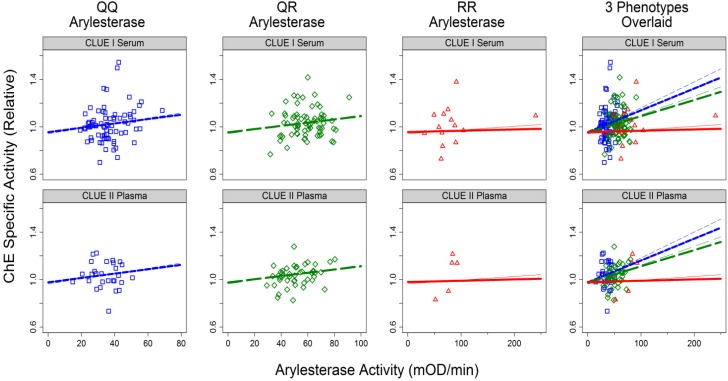
ChE specific activity *vs*. arylesterase activity by sample source (CLUE I *vs*. II) and arylesterase phenotype (QQ, QR, and RR).

**Figure 5:**
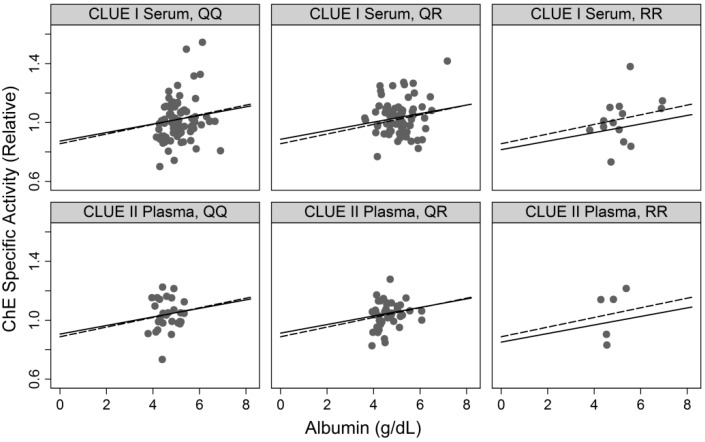
ChE specific activity *vs*. albumin by sample source (CLUE I *vs*. II) and arylesterase phenotypes (QQ, QR, and RR).

The primary aim of this study was to examine association between arylesterase activity and ChE specific activity and determine if the association differs by arylesterase phenotype (QQ, QR, and RR), with or without adjustment for albumin. This analysis was conducted accounting for ChE phenotypes since as described in the preceding section they are strong determinants of ChE specific activity. Figure 4 and Figure 5 show the results graphically with the data from individual samples along with fitted lines based on the two regression models (*i.e.*, with or without adjustment for albumin).

Table 2 shows the results numerically with the estimated regression coefficients from the model with adjustment for albumin as well as other interim models. The partially-adjusted models included terms for ChE phenotypes and sample sources (CLUE I *vs*. II). The fully (simultaneously) adjusted model included terms for ChE phenotypes, sample sources, arylesterase activity (specific to inferred arylesterase genotype), and albumin. Estimated coefficients for arylesterase and albumin in the fully-adjusted model were attenuated compared to those in the partially-adjusted model, and evidence for the association also weakened slightly yet remained strong.

ChE specific activity was positively associated with arylesterase activity for subjects with QQ and QR arylesterase phenotypes, but there was little evidence for such an association for RR phenotype (Figure 4). QQ and RR are homozygotes for alleles with low and high, respectively, turnover number for paraoxon. When adjusted for ChE phenotype and sample source (CLUE I *vs*. CLUE II), increases in ChE specific activity associated with each 50 (mOD/min) increase in arylesterase activity in individuals with QQ, QR, and RR arylesterase phenotype were 0.11 (*p* = 0.004), 0.08 (*p* = 0.001), and 0.02 (*p* = 0.39), respectively. Main effect terms for sample sources and ChE phenotypes were included in the model (and in all models for ChE variables hereafter). Thin and thick lines were fitted without or with, respectively, adjustment for observed association with albumin, which will be discussed later.

Thin and thick lines were fitted using two separate models without or with, respectively, adjustment for albumin. Both models included terms for ChE phenotypes and sample source. Data points for ChE phenotype of UU only are displayed although data for other ChE phenotypes also were used when fitting models. Note that slopes, not intercepts, varied by the inferred arylesterase genotype. Intercepts varied by sample source (0.81 for CLUE I *vs.* 0.85 for CLUE II, *p* = 0.02).

Dashed and solid lines were fitted using two separate models without or with, respectively, adjustment for arylesterase. Both models included terms for ChE phenotypes and sample source. Data points for ChE phenotype of UU only are displayed although data for other ChE phenotypes also were used when fitting models.

**Table 2:** Association between various factors and ChE specific activity.

Variable	Description (unit)	Partially-Adjusted Model ^a^			Fully-Adjusted Model ^b^		
		β	(95% CI)	*p* ^c^	β	(95% CI)	*p*
Arylesterase Activity	Continuous (mOD/min)			0.004			0.007
	For QQ phenotype	0.0022	(0.0007, 0.0037)	0.004	0.0018	(0.007, 0.068)	0.01
	For QR phenotype	0.0016	(0.0006, 0.003)	0.001	0.0014	(0.0004, 0.0023)	0.005
	For RR phenotype	0.0003	(−0.0004, 0.0010)	0.4	0.0001	(−0.0006, 0.0009)	0.76
	Interaction			0.002			0.002
Albumin	Continuos (g/dL)	0.033	(0.010, 0.055)	0.004	0.029	(0.007, 0.051)	0.01
ChE Phenotype	Indicator (unitless)			2 × 10^−16^			9 × 10^−16^
	UA *vs.* UU (reference)	−0.21	(−0.27, −0.15)	4 × 10^−11^	−0.19	(−0.25, −0.13)	5 × 10^−10^
	AA *vs.* UU (reference)	−0.59	(−0.81, −0.37)	0.02	−0.52	(−0.73, −0.31)	1 × 10^−6^
	UF *vs.* UU (reference)	0.15	(0.023, 0.28)	1 × 10^−7^	0.07	(−0.05, 0.20)	0.26
Sample Source	Indicator (unitless)			0.35			0.02
	CLUE II *vs.* I (reference)	0.017	(-0.017, 0.051)		0.037	(0.007, 0.068)	
Intercept	Indicator (unitless)	1.02	(1.00, 1.05)		0.81	(0.69, 0.92)	

^a^ Adjusted for ChE phenotype, sample source; ^b^ Adjusted for ChE phenotype, sample source, arylesterase activity, and albumin; ^c^ All *p*-values based on a Wald test with a null hypothesis that a (group of) coefficient(s) are equal to zero.

Slopes varied by inferred arylesterase genotype either when unadjusted (*p* = 0.002) or adjusted (*p* = 0.002) for albumin. The slope for RR differs from that for QQ (*p* = 0.001 or 0.003, unadjusted or adjusted, respectively, for albumin) while the slope for QR did not differ from that for QQ (*p* = 0.15 or 0.26 unadjusted or adjusted, respectively, for albumin). Figure 4 and Figure 5 show data for subjects with ChE phenotype of UU only although models were fitted to the whole data including those for samples of other ChE phenotypes.

In a partially-adjusted model with arylesterase and ChE phenotype terms (data not shown), there was weak evidence for difference across sample sources (difference = 0.02, *p* = 0.13) but the sample source indicator (CLUE I *vs*. II) was included as it confounded the association between arylesterase and ChE specific activity (the inclusion of the indicator increased the QQ-specific slope for arylesterase activity by 12%). There was little evidence for different intercepts for the three inferred arylesterase genotypes (*p* = 0.74). There was little evidence that the arylesterase coefficients for UU and UA ChE phenotypes were different (*p* = 0.49). The point in the “CLUE I, arylesterase RR” panel with an exceptionally high arylesterase activity was not influential (for testing RR-specific slope = 0, *p* = 0.39 and 0.57 before and after, respectively, inclusion of the indicator for the high point).

Adjustments for albumin did not alter the qualitative conclusions presented. The adjustment attenuated the slopes for arylesterase (e.g., from 0.0022 to 0.0018 for QQ), providing weaker yet still strong evidence for overall arylesterase-ChE specific activity association (*p*-value increased from 0.004 to 0.007).

Albumin was positively associated with ChE specific activity (Figure 5). Dashed and solid lines show linear relationships between the two variables without and with, respectively, adjustment for arylesterase. The adjustment resulted in decrease in the estimated regression coefficients common to all six panes shown (0.033 to 0.029) and provided slightly weaker evidence (*p*-value increased from 0.004 to 0.01). There was little evidence (*p* = 0.71) that genotype-specific arylesterase-ChE specific activity associations differ by sample source.

There was little evidence for association between ChE specific activity and: education; age at blood donation; month of blood donation, or; smoking status (data not shown). In terms of sex difference, ChE specific activity was estimated to be higher for women by 0.011 in a “fully-adjusted” model with a sex term, but the evidence was weak (*p* = 0.54).

While statistical evidence for some of the observed associations are strong as judged by small *p*-values, substantial variation remains unexplained in the fully-adjusted model. The marginal R-squared for the fully-adjusted model was 23.7% with the partical marginal R-squared for arylesterase variables of 1.0%.

### 3.2. Discussion

ChE specific activity had between-sample variation that was not attributed to measurement error as evidenced by good-to-excellent intraclass correlation coefficients. Cholinesterase phenotypes accounted for the variation in the manner expected from known differences in their catalytic efficiency. More importantly, some of the variation was statistically accounted for by association with arylesterase activity, which varied by arylesterase phenotype. ChE specific activity and arylesterase activity were positively correlated, giving support for the hypothesis that arylesterase has *in vivo* detoxifying capacity against ChE inhibiting agents in blood of subjects in general population.

This positive association was strongest in magnitude among individuals with QQ arylesterase phenotype and less marked among QR individuals. QQ and RR are homozygotes for low and high, respectively, paraoxonase activity alleles [23]. The effect of the polymorphism, though, has been shown to be reversed for the *in vitro* hydrolysis of diazoxon, soman and sarin in the presence of high concentration NaCl, thus changing the order of relative protective capacity of isozymes [24]. Nonetheless, in a follow-up animal experiment Q and R PON_192_ alleles were shown: to have similar diazoxonase activity in physiological condition with low NaCl concentration and; to provide similar protection against diazoxon challenge [25]. In our study, among individuals with phenotypes of higher arylesterase activity (corresponding to QR or RR) association between arylesterase activity and ChE specific activity was weaker in magnitude than among QQ individuals. It can be speculated that cholinesterase inhibitors contributing to the ChE inhibition in our subjects were probably not paraoxon, chlorpyrifos oxon or other OPs that are preferentially hydrolyzed *in vivo* by high activity paraoxonase isozyme. Further we can speculate that cholinesterase inhibitors that contributed to the arylesterase-ChE specific activity association did not change between 1974 and 1989 in terms of whether they were preferentially degraded by PON1_192_ Q or R isozyme as the arylesterase phenotype-specific arylesterase-ChE association did not differ between 1974 and 1989 samples.

In contrast to our findings, PON1_192_ Q, not R, was associated with greater ChE inhibition in a recent study using ChE monitoring data for pesticide handlers [26]. In that study, association between arylesterase genotype/activity and differences in serum butyrylcholinesterase activity measured at pre- and mid-application periods were investigated. They found that, similar to our results, within each arylesterase genotype, greater ChE inhibition was associated with low arylesterase activity. Consistent with the fact that R alloform has greater catalytic capacity towards paraoxon, previous epidemiological and experimental studies tended to show PON1_192_ R alloform was more protective, but some studies have shown the association in the opposite direction. For instance, Mackness *et al*. [27] found that PON1_192_ R was associated with greater toxicity. It is notable most previous epidemiological investigations involved occupationally-exposed individuals while in the present study subjects were drawn from a general population. Note also that raw ChE activity was used as the endpoint by Hofmann *et al*. [26], meaning that the association they found may actually represent that between arylesterase genotype/activity and ChE mass in serum. An implicit assumption that ChE mass was unchanged between pre- and mid-application periods was made when interpreting lower ChE activity as greater inhibition.

The interpretation described above for the observed regression coefficients is based on the assumption that arylesterase *activity* was the relevant quantity for assessing the association. Alternatively, arylesterase *mass* concentration could have been the biologically more relevant covariate. We did not measure arylesterase mass, but our activity measurements could be converted to quantities that can be interpreted as relative mass concentrations using conversion factors proposed by Richter *et al*. [28]. This is possible through converting our arylesterase activity, which was measured in the presence of 2 M NaCl, to diazoxonase activity in physiological condition since PON1_192_ Q and R isozymes hydrolyze diazoxon at similar rates under physiological conditions. To achieve this conversion, arylesterase activities for PON1_192_ QR and RR subjects we measured were multiplied by 1.19 and 1.69, respectively [28]. Converted QR and RR arylesterase activity could be interpreted as relative arylesterase mass concentration expressed as equivalent QQ arylesterase activity. A fully-adjusted regression model shown in Table 2 was refitted to the QQ equivalent arylesterase activity data. The coefficients for converted QR and RR arylesterase activity were 0.0012 (*p* for non-zero = 0.005) and 0.00007 (*p* = 0.76), respectively. Difference between the coefficients for converted QQ activity (0.0018) and for converted QR activity (0.0012) was not statistically significant (*p* = 0.12). This is similar to the corresponding finding obtained on an activity basis (*p* = 0.26) described earlier. The statistical evidence for heterogeneity of arylesterase activity coefficients across arylesterase genotypes remained strong (*p* = 0.002, which is unchanged from per-activity analysis since the two analyses differed only on scale). The qualitative conclusion that the increasing amount of arylesterase of PON1_192_ Q genotype, but not that of R genotype, appears to provide protection is unchanged whether we perform analysis using arylesterase activity or mass equivalent. Of note, we did not determine actual PON1_192_ genotype and used phenotyping results based on a functional assay involving a differential inhibitor. Nonetheless, phenotypic separation in Figure 1 was comparable to those obtained using some of the dual substrate assays, which have been used widely and recommended for determining “PON1 status” (*i.e.*, simultaneously ascertaining phenotypes corresponding to PON1_192_ genotypes and measuring paraoxonase activity levels [9]) in a simple and speedy manner.

The interpretation of these results as metabolic protection conferred by arylesterase is based on the assumption that the subjects we studied should have some exposure to cholinesterase-inhibitors that are detoxified by arylesterase, such as organophosphates and carbamates. As mentioned in the Introduction, exposure to these agents is and would have been ubiquitous in the US general population. Yet, that does not necessarily mean that the level of such exposure is high enough to result in noticeable inhibition in plasma cholinesterase.

A recent risk assessment study by Payne-Sturges *et al*. [29] has some relevance on this issue. They assessed whether exposure to common organophosphates in the general population would cause inhibition in brain acetylcholinesterase using urine metabolite data from the National Health and Nutrition Examination Survey (NHANES) conducted in 1999–2002. Their results indicate that even the observed high-end body burden levels (95th percentile) for adults is 1/100 or lower than the level associated with 10% decrease in brain acetylcholinesterase activity. These results are in contrast to our observation where about 1/6 of samples had greater than 10% inhibition although a direct comparison between the two studies is difficult. In the present study, we measured blood butyrylcholinesterase, which sometimes is more inhibited by some organophosphates (e.g., chlorpyrifosoxon) than acetylcholinesterase. Also organophosphate body burden profiles could be substantially different across sampling time points (1974 and 1989 for our study *vs*. 1999–2002 for NHANES) and location (Western Maryland for our study *vs*. nationwide for NHANES). Payne-Sturges *et al*. [29] *estimated*, rather than *measured* as we did, cholinesterase inhibition levels based on many assumptions. Nonetheless, assuming some similarities in body burden levels across time and location, it seems unlikely that the common organophosphates were responsible for greater than 10% inhibition seen in our samples. We may infer that some cholinesterase inhibitors other than the common organophosphates accounted by Payne-Sturges *et al*. [29] could have been contributors in the variation in specific ChE activity observed in the present study.

Subjects included in our study were potentially exposed to ChE inhibiting insecticides through home gardening, residential pest control, residues in food, flea control devices for pets, *etc*., most of which would have been reflected in the analysis by Payne-Sturges *et al*. [29] although they did not consider carbamates or other classes of cholinesterase inhibitors. Our ChE activity assay was likely to be sensitive enough to reflect ChE inhibition occurring *in vivo* in subjects that were caused by carbamates and other cholinesterase inhibitors including naturally-occurring ones. Many organochlorine insecticides were phased out since the 1970’s, and less persistent OPs and carbamates were used increasingly in the 1980’s for indoor use. Participants in the 1989 CLUE II study may have had greater potential for exposure to the OP/carbamate compounds used for residential pest control than participants in the 1974 CLUE I study. In our data, though, the levels of ChE inhibitions were slightly greater for 1974 samples than 1989 samples. This change could be explained by the aforementioned general decline in overall organophosphates use, which includes not only indoor residential use but also agricultural application that results in exposure through diets. Some carbamates are used as prophylaxis against OP exposure or medications. These probably represented minor uses among our subjects and are unlikely to explain residual variation in ChE specific activity.

We are unaware of any common environmental agents that enhance cholinesterase activity. From this we infer the presence of cholinesterase inhibitors, not enhancers, in sera/plasmas would be the main source of variation in ChE specific activity. If so, we would expect to see a left-skewed distribution for the ChE specific activity. Distributions for specific activity actually were: right-skewed for CLUE I sera; and symmetrical for CLUE II plasmas.

The positive association between ChE specific activity and arylesterase activity might be explained by mechanisms unrelated to cholinesterase inhibitor metabolism. The positive association could be explained by presence of certain circulating bioactive substance(s) with the effects of stimulating both arylesterase and cholinesterase. Some *in vitro* studies have shown that certain phosphatidylcholines increase arylesterase activity [30]. Liposome-forming phosphatidylcholines also increase activity of horse cholinesterase [31]. These observations collectively indicate that serum ChE specific activity levels could be correlated with serum paraoxonase activity because certain plasma lipids may stimulate both of these activities. However, when we assessed *in vitro* effects of phosphatidylcholine and related phospholipid compounds on ChE specific activity, none showed the anticipated effect of elevating ChE activity (data not shown). For the observed PON1_192_ genotype-specific arylesterase-ChE association to arise, stimulatory effects of plasma lipids also would need to be PON1_192_ genotype-specific. PON1_192_ Q isozyme was found to have lower affinity with HDL-apoA-I [32]. Binding of arylesterase with HLD-apoA-I was reported to increase its stability and activity [33]. The association between PON1_192_ Q arylesterase activity and ChE specific activity that we observed may be explained by yet-to-be-identified plasma lipid component(s) that: facilitate binding of low-affinity PON1_192_ Q, not R, isozyme with HDL-apoA-I; and stimulate ChE activity.

Certain lipid-lowering medications also may have dual-stimulating capacity. For instance, fenofibrate increases paraoxonase activity [34] and increases ChE specific activity [35]. Clofibrate increases ChE activity [36], yet there have been inconsistent findings as to its effects on plasma paraoxonase activity [36,37]. Fenofibrate would not have been taken by subjects in the current study as the drug was first marketed widely in the U.S. in 1998 [38], almost a decade after the last collection of the blood samples used in the current study. About a half of the blood samples used in the current study were collected in 1974, at which time some lipid-lowering medications including clofibrate were in use for treating hyperlipidemia in the U.S. Taken together, we cannot exclude the possibility that the observed positive association between arylesterase activity and ChE specific activity is attributable at least in part to the use of clofibrate in some of our subjects. This explanation, however, does not clarify why the positive association is mostly limited to subjects with arylesterate phenotype associated with PON1 192 Q allele.

A number of human observational studies have reported strong associations between serum paraoxonase activity and serum lipids [39,40,41,42] while others have reported that ChE activity was elevated with conditions characterized by elevated serum lipid levels such as hyperlipidemia, diabetes, and obesity [43]. It is worth discussing how these conditions, which often coexist, might operate as a confounder in the association between arylesterase and cholinesterase and whether it is necessary to adjust for them in the examination of the association. For any of these conditions to be a genuine confounder it has to be associated with arylesterase and is a causal factor for changes in ChE specific activity. If it were in the causal pathway between arylesterase and ChE specific activity, then we have a reason not to treat it as a confounder. In brief, various findings to date reviewed below indicate that the observed positive association between arylesterase and ChE specific activity is unlikely to have arisen through confounding due to the aforementioned conditions characterized by elevated lipid levels.

First, we consider the association between serum lipid levels and paraoxonase activity. Insulin-dependent and -independent diabetes mellitus are associated with reduced paraoxonase activity in most studies, and the reduction is thought to result from greater glycation of paraoxonase among diabetics [44]. Many studies have reported that hyperlipidemia is accompanied by lower paraoxonase activity [45,46]. The question as to whether hyperlipidemia causes low paraoxonase activity or vice versa (or there is a common cause for both) has not been completely settled. Some clinical trials, however, demonstrated paraoxonase-increasing capacity of some lipid-lowering medications [34], rendering some credibility to the view that hyperlipidemia is the cause, not the consequence, of the low paraoxonase activity. Taken together, changes in paraoxonase/arylesterase activity can arguably occur as a result of diabetes and hyperlipidemia, and if that is the case adjustment for these conditions would be unnecessary in the assessment of association between arylesterase activity and ChE specific activity.

Next we consider the association between ChE specific activity and those conditions characterized by elevated serum lipids. As mentioned earlier, these conditions typically were accompanied by higher ChE activity as well. Various mechanisms to account for the positive association have been proposed to date. They can be grouped into four types, which would lead to different conclusions regarding the need to adjust for the condition. 

We have mentioned the type of explanation involving certain yet-to-be-identified circulating bioactive substances, the presence of which in plasma stimulates ChE specific activity. The second explanation stipulates that those conditions associated with higher serum lipids cause certain physiological changes in ChE synthesis such that tertiary and/or quaternary structure of ChE enzyme is altered resulting in the increase in specific activity. If these mechanisms are in fact operating, then it would be justified to adjust for those conditions in our analysis on arylesterase-ChE association. Yet, such an adjustment would result in an enhancement, rather than the attenuation, of the magnitude of the observed positive association because those conditions are generally negatively associated with paraoxonase activity in plasma.

The third type of explanation, which is the earliest published one, stipulates that these conditions affect the hepatic metabolism, increasing synthesis of ChE enzyme [47] or decreasing degradation of ChE thereby increasing plasma concentration of ChE. If this were the case, there would have been no need to adjust for diabetes mellitus in our analysis as the ChE specific activity, our main outcome, would have remained unaffected by these conditions.

The fourth explanation assumes the causal role of higher ChE activity in the development of certain lipid abnormalities. Such an explanation is supported by the observation that administration of ChE inhibitor could reverse hypertriacylglycerolemia induced chemically through a non-ChE dependent mechanism in mice [43]. If specific ChE activity is operating such a causal role in our data, it is not necessary to adjust for diabetes in our analysis: generally speaking there is no need to adjust for the events that occur as a result of changes in the dependent variable, which is ChE specific activity in our analysis.

In summary, in the presence of certain presumed causal associations there is no need to adjust for the conditions reportedly accompanied by higher ChE activity. In the presence of other presumed causal associations such an adjustment would be warranted, yet the adjustment, if performed, would have resulted in observing more accentuated positive associations than actually observed. Overall, there is little basis to attribute the observed associations to the confounding due to those conditions. We have not performed adjusted analysis since our capacity to perform the adjustment was limited: for the majority of the subjects in this study detailed information on health conditions such as diabetes and hyperlipidemia were not collected at the time of participation.

Our observation regarding arylesterase and cholinesterase also needs to be scrutinized in the context of enzyme induction/suppression by environmental and behavioral factors. Observational studies indicate that plasma paraoxonase levels are negatively correlated with vegetable intake [48], and are not correlated [49] or positively correlated [50], with intake of vitamins C and E. In human intervention studies, serum paraoxonase levels are decreased with high vegetable diets [51] but are increased with consumption of antioxidant-rich pomegranate juice [52]. As such, findings on effects of antioxidant-rich diets on serum paraoxonase are apparently inconsistent, but the observational [48] and intervention [51] studies from Finland both have shown high vegetable intake could decrease paraoxonase level. Such a negative association might explain the positive association between paraoxonase and ChE specific activity we observed if vegetables consumed by our subjects had paraoxonase-decreasing effects and also contained sufficiently high concentrations of naturally-occurring cholinesterase inhibitors, e.g., solanine in potatoes [53].

The observed *positive* association between ChE specific activity and albumin was unexpected. Due to its potential to sterically hinder substrate’s access to ChE active site [54] we anticipated that albumin may have inhibitory effects on ChE specific activity, producing a *negative* association. The positive association between ChE specific activity and albumin could arise if albumin had effects of either increasing the ChE activity measurement or decreasing the ChE mass measurement. The former may be due to molecular interaction among ChE, albumin and free fatty acid: Sakoguchi *et al*. [55] reported that free fatty acids had suppressive effects on ChE activity *in vitro*, which was abolished by addition of albumin presumably due to albumin’s capacity to sequester fatty acids. The latter would occur if albumin interfered with binding of the primary antibody used in the ELISA for ChE mass measurement. This second possibility was minimized by the use of a monoclonal, which is less likely to cross-react with albumin than often-used polyclonals, as a catchment antibody in our ELISA for ChE mass.

A multivariate fitted line describing albumin-ChE specific activity association had different intercepts for CLUE I sera and CLUE II plasmas (the intercept was higher by 0.037 for CLUE II than for CLUE I, *p* = 0.02). In analyses not adjusting for albumin, however, the specific activity was not different between these two groups. This contrast was due to the tendency of albumin measurements to be higher for CLUE I than for CLUE II. This observation is consistent with a previous report [56] that storage at −25 °C of sera induced an apparent increase proportional to storage time in albumin concentration determined by the bromocresol green method, which is similar to the bromocresol purple albumin assay we employed. CLUE I samples had been stored for 29 years, about twice as long as 14 years of storage for CLUE II samples. The normal range for serum/plasma albumin is 3.5–5.0 g/dL [57]. The means of albumin measurements were 5.1 g/dL for CLUE I sera (55% of which had abnormally high albumin, *i.e.*, >5 g/dL) and 4.6 g/dL for CLUE II plasmas (24% of which had albumin > 5 g/dL); the CLUE I *vs*. II difference was highly significant (*p* = 6 × 10^−11^). Thus we conclude the observed elevation in albumin may be due to an assay-specific storage artifact.

An indicator for sample source (CLUE I *vs*. II) was included in the various GEE models as it was statistically significant in the fully-adjusted model and confounded certain associations. This indicator represents a combination of many factors that can potentially influence our laboratory measurements, including serum *vs*. plasma, sample collection timing (1974 *vs*. 1989), sample storage time (29 *vs*. 14 years), and other possible differences in sample collection or handling practices across two studies. The longer storage time, which could result in greater deterioration of ChE enzyme, and/or aforementioned likely decline in exposure to ChE-inhibiting insecticides across the two time points of sample collection may account for the lower ChE specific activity of CLUE I sera.

There was much variation in ChE specific activity even after accounting for associations with the covariates we considered. The residual variation is unlikely to be explained entirely by measurement error, judging from the satisfactorily high intraclass correlation coefficient of 0.78 for ChE specific activity. This meant that about 80% of the total variation represented variation across samples and about 20% measurement error.

How much of the observed variation in ChE specific activity was attributable to exposure to man-made cholinesterase inhibitors or other modulating factors is unknown. To better evaluate this question, simultaneous assessment of exposure to cholinesterase and blood ChE specific activity would be necessary. Two studies have taken such an approach using a crude exposure grouping [4,5]. Kotani *et al*. found two sarin poisoning victims had only 14% and 25% of serum ChE activity expected from their serum ChE mass concentrations. Brock (1991) found that ChE specific activity levels are not associated with OP exposure among insecticide factory employees. The mass ELISA assay by Brock *et al*, though, might have suffered from the aforementioned assay artifact attributable to a polyclonal used as the catchment antibody. We implemented measures to ameliorate such an assay artifact and to minimize ChE reactivation, which also can potentially obscure any decrease in ChE specific activity due to exposure to cholinesterase inhibitors.

Our findings of no association between a few investigated factors and ChE specific activity fill some knowledge gaps left by previous studies, which predominantly evaluated raw ChE activity only, rather than specific activity. In these studies changes in raw ChE activity was often assumed to have occurred as a result of changes either in ChE specific activity or ChE mass, depending upon available extraneous supporting information. Males were found to have higher ChE activity than females [58,59], possibly due to greater alcohol intake among men. We did not observe any sex-related difference in ChE specific activity; this suggests the previously observed associations could have been due to sex-related differences in ChE mass. In fact, males had slightly higher ChE mass than females in the present study although the evidence was not strong (data not shown). Oral contraceptive use and pregnancy are associated with decreased activity, indicating some hormonal control of ChE synthesis/degradation [2]. These decreases are thought to result from reduction in hepatic ChE synthesis and so the variation would be due to a decrease in ChE mass, not specific activity. Older age in adulthood was associated with higher ChE activity in one study [58], but not in another [59]. ChE activity was found to be decreased in summer months in one study [60] and the authors speculated the decrease was due to increased exposure to residues of ChE inhibiting insecticides in fruits and vegetables. In the present study we observed no seasonal variation in ChE specific activity (neither in ChE mass nor ChE raw activity, data not shown).

The present investigation used pseudocholinesterase, not red blood cell acetylcholinesterase, mainly for logistic reasons; red blood cells were available only from CLUE II. In the future it would be worthwhile to investigate the utility of acetylcholinesterase specific activity as an exposure marker.

## 4. Conclusions

We observed associations consistent with protective effects of arylesterase against ChE inhibition. This adds to the growing evidence that a certain arylesterase phenotype, either having a particular isozyme(s) or having low quantities of enzyme in blood, manifests itself as a susceptibility factor for intoxication by OPs. We also found that ChE specific activity is positively correlated with serum albumin. Further validation and characterization of the assay of ChE specific activity as a biomarker of exposure to ChE inhibitors are warranted, especially investigation into the contribution of various factors to within- and between-subject variation of ChE specific activity in longitudinal studies.
